# Rapid triage for ischemic stroke: a machine learning-driven approach in the context of predictive, preventive and personalised medicine

**DOI:** 10.1007/s13167-022-00283-4

**Published:** 2022-05-27

**Authors:** Yulu Zheng, Zheng Guo, Yanbo Zhang, Jianjing Shang, Leilei Yu, Ping Fu, Yizhi Liu, Xingang Li, Hao Wang, Ling Ren, Wei Zhang, Haifeng Hou, Xuerui Tan, Wei Wang, on behalf of Global Health Epidemiology Reference Group (GHERG)

**Affiliations:** 1 Centre for Precision Health, Edith Cowan University, 270 Joondalup Drive, Joondalup, 6027 Western Australia Australia; 2 The Second Affiliated Hospital of Shandong First Medical University, Tai’an, Shandong China; 3 Dongping People’s Hospital, Tai’an, Shandong China; 4 Tai’an City Central Hospital, Tai’an, Shandong China; 5 Ti’men Township Central Hospital, Tai’an, Shandong China; 6 School of Public Health, Shandong First Medical University & Shandong Academy of Medical Sciences, 619 Changcheng Road, Tai’an, 271016 Shandong China; 7 Department of Clinical Epidemiology and Evidence-Based Medicine, National Clinical Research Centre for Digestive Disease, Beijing Friendship Hospital, Capital Medical University, Beijing, China; 8 Beijing Key Laboratory of Clinical Epidemiology, School of Public Health, Capital Medical University, Beijing, China; 9 Beijing United Family Hospital, No.2 Jiangtai Road, Chaoyang District, Beijing, China; 10 Centre for Cognitive Neurology, Department of Neurology, Beijing Tiantan Hospital, Capital Medical University, Beijing, China; 11 The First Affiliated Hospital of Shantou University Medical College, Shantou, Guangdong China; 12 Institute for Nutrition Research, Edith Cowan University, Joondalup, WA Australia

**Keywords:** Predictive preventive and personalised medicine (PPPM/3PM), Ischemic stroke, Machine learning, Objective clinical data, Disease prediction, Targeted prevention, Patients stratification, Improved individual outcomes

## Abstract

**Background:**

Recognising the early signs of ischemic stroke (IS) in emergency settings has been challenging. Machine learning (ML), a robust tool for predictive, preventive and personalised medicine (PPPM/3PM), presents a possible solution for this issue and produces accurate predictions for real-time data processing.

**Methods:**

This investigation evaluated 4999 IS patients among a total of 10,476 adults included in the initial dataset, and 1076 IS subjects among 3935 participants in the external validation dataset. Six ML-based models for the prediction of IS were trained on the initial dataset of 10,476 participants (split participants into a training set [80%] and an internal validation set [20%]). Selected clinical laboratory features routinely assessed at admission were used to inform the models. Model performance was mainly evaluated by the area under the receiver operating characteristic (AUC) curve. Additional techniques—permutation feature importance (PFI), local interpretable model-agnostic explanations (LIME), and SHapley Additive exPlanations (SHAP)—were applied for explaining the black-box ML models.

**Results:**

Fifteen routine haematological and biochemical features were selected to establish ML-based models for the prediction of IS. The XGBoost-based model achieved the highest predictive performance, reaching AUCs of 0.91 (0.90–0.92) and 0.92 (0.91–0.93) in the internal and external datasets respectively. PFI globally revealed that demographic feature age, routine haematological parameters, haemoglobin and neutrophil count, and biochemical analytes total protein and high-density lipoprotein cholesterol were more influential on the model’s prediction. LIME and SHAP showed similar local feature attribution explanations.

**Conclusion:**

In the context of PPPM/3PM, we used the selected predictors obtained from the results of common blood tests to develop and validate ML-based models for the diagnosis of IS. The XGBoost-based model offers the most accurate prediction. By incorporating the individualised patient profile, this prediction tool is simple and quick to administer. This is promising to support subjective decision making in resource-limited settings or primary care, thereby shortening the time window for the treatment, and improving outcomes after IS.

**Supplementary Information:**

The online version contains supplementary material available at 10.1007/s13167-022-00283-4.

## Introduction

### Ischemic stroke is a major cause of death and disability globally

Stroke is one of the leading causes of morbidity and mortality worldwide, and the risk factors for stroke are complicated, such as cardiovascular diseases, diabetes, hyperlipidaemia, and unhealthy lifestyles [1, 2]. Ischemic stroke (IS) accounts for approximately 87% of all stroke cases: ischemic stroke, haemorrhagic stroke, and transient ischemic attack [3]. In China, stroke became the top leading cause of years of life lost, with rising mortality rates from 106 per 100,000 persons in 1990 to 149 per 100,000 persons in 2017 [4]. China national report showed that the age-standardised prevalence of stroke reached 1114.8 per 100,000 persons in 2013, imposing an enormous burden on the healthcare system [5]. In middle-income countries, only 10% to 20% of stroke patients could reach the hospital within 3 h (treatment during this period may still lead to disability). From the perspective of predictive, preventive and personalised medicine (PPPM/3PM), a prompt and accurate diagnosis of the stroke allows for reducing treatment delay and improving stroke outcomes [5].

### Challenges in triaging patients with ischemic stroke

For now, the diagnosis of stroke in the less developed area mainly relies on neurological examination. However, this physical examination performed by a less experienced examiner can result in diagnoses with lower accuracy and reliability [6]. Moreover, the reported prediction models for IS diagnosis mostly relied on the conventional statistical models. For example, Cox proportional hazard model uses selected features for the prediction of disease occurrence, which is hard to predict discrete events and has a relatively low efficiency [7, 8]. Therefore, to improve subjective decision making in resource-limited settings, a paradigm change from reactive medicine to PPPM/3PM is needed [9]. We, therefore, developed and validated the predictive tool of IS using individualised IS patient profiles, and we also reported its feasibility.

### Machine learning is an optimistic strategy for ischemic stroke diagnosis in the context of PPPM/3PM

In the context of PPPM/3PM, the real-time predictive analytic tool of IS can be instructive in identifying those at high risk who may benefit from the prompt intervention e.g. thrombolysis with alteplase and endovascular treatment [10–12]. Artificial intelligence (AI) approaches can incorporate high dimensional and multivariate data to solve these challenging issues [13], and machine learning (ML) is a subdomain of AI involving the automatic discovery of patterns within data [14]. Among various ML-based models, supervised learning tools, e.g. random forest, neural network, and extreme gradient boosting (XGBoost), can learn complicated structures by incorporating numerous variables with multiple dimensional data [13, 15]. Furthermore, owning to the outstanding predictive performance [16], ML approaches have been applied to solve real problems in the framework of PPPM/3PM, including predictors selection, predictive diagnostics, targeted prevention, and personalised medical services [9, 17, 18].

Although complex ML models provide high prediction accuracy, they are less human-understandable. For critical applications in the field of medicine, explanations of ML-based prediction models are essential for users to understand and trust the models established [19]. Extra techniques to peer into the black-box ML models are thus needed. Permutation feature importance (PFI) is a global explanation method that provides insights into the model’s behaviour in general [20]. Apart from global explanation, local interpretable model-agnostic explanations (LIME) and SHapley Additive exPlanations (SHAP) are two well-accepted local explanation approaches to interpret why a certain prediction was made for a specific individual by incorporating the individualised patient profile [21].

### Working hypothesis

**Fig. 1:**
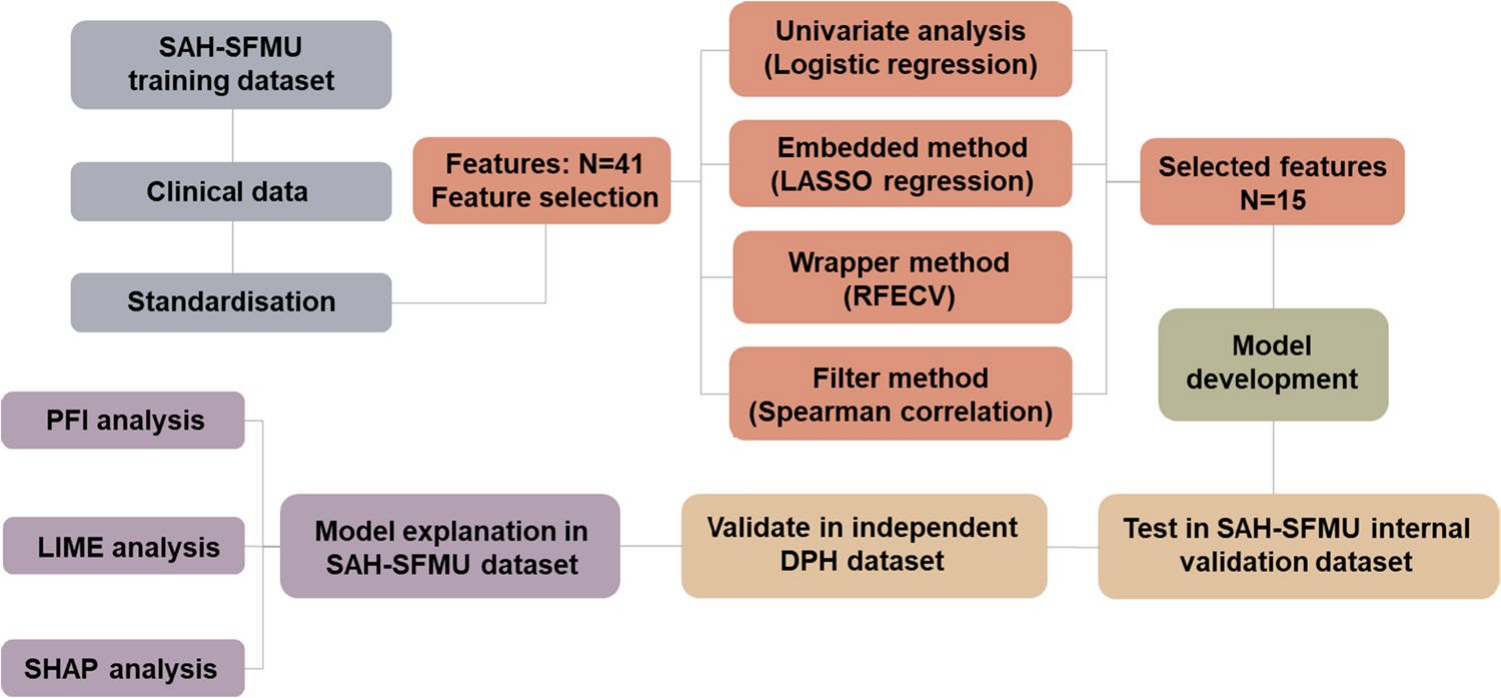
Schematic diagram overview of the study. The overview illustrates five primary processes: data acquisition, feature selection, model development, model validation, and model explanation. SAH-SFMU, Second Affiliated Hospital of Shandong First Medical University; LASSO, least absolute shrinkage and selection operator; RFECV, recursive feature elimination with fivefold cross-validation; DPH, Dongping People’s Hospital; PFI, permutation feature importance; LIME, local interpretable model-agnostic explanations; SHAP, SHapley Additive exPlanations

We aimed to develop and validate an ML-based prediction tool for quickly and accurately triaging the patients with risk of IS in the framework of PPPM/3PM. We hypothesised that supervised ML classification algorithms may yield better discrimination between individuals with and without IS than that of conventional statistical models. Therefore, in this current study, we used cost-effective clinical laboratory features to develop and validate ML-based models for the classification of IS and employ interpretation methods for explaining black-box ML models (Fig. 1).

## Methods

### Datasets

For developing the ML-based models, a derivation dataset of 10,476 subjects (with 4999 IS-present patients and 5477 IS-absent controls) was used. The participants were obtained from the Second Affiliated Hospital of Shandong First Medical University (SAH-SFMU) between January 2015 and December 2019. Clinical data were obtained from the electronic medical records (EMRs), and the data collection was performed after the reference test (computed tomography [CT] and/or magnetic resonance imaging [MRI]) and the index test (routine haematological and biochemistry tests). The outcome IS (ICD-11, 8B11) was defined as a sudden symptom of neurological deficit (i.e., sudden weakness, numbness, lessened control of one side of the body, sudden dimness, loss of vision in one or both eyes, loss of speech, dizziness, unsteadiness or sudden fall, and difficulty in wallowing), followed by the diagnosis of CT angiography and/or MRI, which are the prerequisite conditions to avoid delaying thrombolytic therapy [22].

To evaluate the performance of the ML-based models, an external validation dataset of 3935 adults was used from another hospital Dongping People’s Hospital (DPH): 1076 patients diagnosed with IS, and 2859 IS-absent controls. Participants were included from December 2019 to October 2021. Demographic and clinical data (routine haematological parameters and common biochemical analytes) were collected. Both SAH-SFMU and DPH hospitals are urban-based healthcare providers. IS-absent controls in this dataset included the individuals who had undergone regular health examinations or presented IS mimics-related diseases or disorders (such as type 2 diabetes, cardiovascular diseases, cancer, headache, dizziness, and limb numbness) at the same hospital, this was analogous to the controls in the SAH-SFMU dataset.

The inclusion criteria of the participants were the following: (1) Chinese Han ethnicity, (2) aged 40–80 years old, and (3) without a history of a diagnosis of IS. Exclusion criteria were the following: (1) pregnant or lactating women, (2) participants with severe mental disorders, and (3) individuals with other serious physical illnesses or injuries. There were no adverse events reported related to the reference test (CT and/or MRI) or the index test (routine haematological and blood biochemistry tests).

The study protocol was approved by the Clinical Ethics Review Committee of the SAH-SFMU (No. 2020–066) and the Clinical Ethics Review Committee of the DPH (No. DPH-06102021). This retrospective study used de-identified patient data and met the criteria for IRB Waiver of Consent Guidance (45 CFR 46.116) [23]; it was therefore permissible to waive the informed consent in this research.

### Sample size

Since there is no generally accepted method to estimate the sample size requirements for a derivation study of the risk prediction model, all accessible data were used to maximise the power and generalizability of results [24]. The reliability of the ML-based model was further examined by exploring an external validation dataset.

## Machine learning methods and statistical analyses

### Data pre-processing

For the derivation dataset, listwise deletion was used to omit those samples with missing data (*n* = 95, the percentage of samples with missing data < 10%). For the external validation dataset, missing data were imputed with the mean of the corresponding feature (continuous distribution) [25].

Feature standardisation was applied to process the numerical values of different scales aiming to improve model performance [26] (Eq. 1):

1 $${x}^{*}=\frac{x-\mu }{\sigma }$$

where the data (*x*) is centralised based on the mean (*μ*) and scaled on the basis of standard deviation (*σ*); the standardised data will follow a distribution with a mean of 0 and a variance of 1.

### Feature selection

Four accepted feature selection methods were applied to the training set based on the free and open-source Python packages: (1) univariate logistic regression [27]; (2) the least absolute shrinkage and selection operator (LASSO) regression (https://scikit-learn.org/stable/modules/generated/sklearn.linear_model.Lasso.html); (3) the recursive feature elimination with fivefold cross-validation (RFECV) (https://scikit-learn.org/stable/modules/generated/sklearn.feature_selection.RFECV.html); (4) the Spearman correlation (https://docs.scipy.org/doc/scipy/reference/generated/scipy.stats.spearmanr.html).

For univariate logistic regression, the variables with *P* < 0.05 were considered the significant predictors for IS. For LASSO regression, according to the regulation weight, the features with non-zero regression coefficients were thought to be IS-relevant predictors. RFECV is a wrapper-type feature selection method; in this study, a support vector classifier was used in core and was wrapped by RFE for helping select features. Spearman correlation determined the direction and the strength of the monotonic relationship between the outcome IS and the feature, features with correlation coefficient > 0.1 in this study were selected.

To improve the ML-based models’ stability and minimise the risks of overfitting, the smallest number of features identified in the SAH-SFMU training set were selected to build the models.

### Model development and validation

A sample of 10,476 participants in the derivation dataset were randomly allocated into a training set of 8380 (80% of the total) and an internal validation set of 2096 (20% of the total). This 80/20 split aimed to permit sufficient training data to quantify the complexity of the model while maintaining adequate data to internally validate the model.

The training set was used to train the ML-based models and tune their corresponding parameters. In the training set, different parameter combinations were exhausted by the grid search algorithm, to determine which set of parameters could achieve the best performance. For each set of model parameters, 9/10 of data were used for fitting the model in turn, and 1/10 of data was used for validation and then repeated 10 times. The area under the receiver operating characteristic (ROC) curve (AUC) was selected as the score of the current parameter combination during the searching process [28]. The internal validation dataset was used to test the established ML-based models on the unseen dataset.

Six ML classifiers—extreme gradient boosting (XGBoost), random forest (RF), neural network (NN), logistic regression (LR), Gaussian Naive Bayes (GaussianNB), and k-nearest neighbours (k-NN)—were employed to generate six prediction models of IS [15, 29–35]. In supervised learning, these models were chosen because ML algorithms can increase the probability of good discriminative performance. Moreover, the usefulness of six ML-based models has been widely reported in medical applications [24, 36]. Detailed elucidations for model establishment are provided in the Supplementary Information.

Model performance was evaluated mainly by AUC, which calculates the area under the ROC curve showing the true positive rate (sensitivity, recall) against the false positive rate (1-specificity) for various threshold values. 95% confidence intervals of AUC, sensitivity and specificity were provided to assess the variability in estimates. Additionally, for the purpose of completeness, other metrics were also performed to evaluate the performance of ML-based models in this investigation, such as classification accuracy, precision score, F1 score, and log loss [31]. Log loss was calculated to indicate the confidence of the prediction. The lower the log loss value is, the lower the uncertainty and the better prediction of the model are for the classification results [37]. Illustrations for the ways and metrics of model evaluation are provided in the Supplementary Information.

To achieve higher generalizability of the model, we applied fivefold stratified cross-validation to obtain the average value of the performance metrics in the training, internal validation, and external validation datasets.

### Model explanation

Explainable AI methods involving global and local methods were applied in the derivation dataset. To explain the ML-based prediction model globally, PFI was ranked based on the relative importance score of each feature (the higher the score is, the more important the feature is to the prediction model). In this study, *accuracy* was chosen as the basis of the importance score for classification [38]. To reveal the impact of input features for a single sample or an individual prediction, local explainable methods (LIME and SHAP) were implemented. The LIME method assumes that the complex ML model is linear on a local scale and verifies the possibility of fitting the simple surrogate model around a specific sample that will mimic how the global model behaves at that locality [21]. SHAP values were estimated based on the XGBoost Tree SHAP algorithm in this study and were presented as log odds ratio for the binary classification task in this study [39].

### Statistical analyses

Continuous variables were reported as median (IQR), and categorical variables were presented as count (%). The Kolmogorov-Smirnoff test was used to verify the normality of the data. Mann–Whitney *U*-test and chi-squared test were performed for continuous and categorical variables respectively. A two-sided *P* < 0.05 was considered statistically significant.

The analyses were performed using Python (version 3.8.5 in Jupyter Notebook) and SPSS (version 25.0, IBM Corporation, Armonk, NY, USA).

### Implementation of the web tool for the triage of IS

To implement the ML-based model into clinical practice, we designed and established an Automatic System for the Triage of Ischemic Stroke (ASTIS) based on those aforementioned ML-based models with the best sensitivity and specificity. The haematological and biochemical predictors were embedded in the web-based tool. User data interaction and visualisation of analysis results were displayed using Nginx, HTML JavaScript, and Flask (Python version).

## Results

### Demographic and clinical characteristics

The demographic and clinical characteristics of the study population are presented in Table 1. In the derivation dataset, 4999 were IS-present patients (47.7%), with similar proportions of patients with IS in the training set (47.3%) and internal validation set (49.5%). In the external validation dataset, 1076 (27.3%) patients had IS occurrence. For data pre-processing in the exploratory data analyses, principal component analysis plots for the derivation and external validation datasets are shown in the Supplementary Information (Fig. S1).

**Table 1:** Characteristics of Participants

		SAH-SFMU Dataset			DPH Dataset		
	Reference interval	IS present	IS absent	*P*-value	IS present	IS absent	*P*-Value
		(*n* = 4999)	(*n* = 5477)		(*n* = 1076)	(*n* = 2859)	
Demographic characteristics							
Age		63.0 [56.0, 70.0]	53.0 [47.0, 61.0]	< 0.001	67.0 [60.0, 74.0]	56.0 [48.0, 66.0]	< 0.001
Sex (count, %)							
Male		2932 (58.7%)	3304 (60.3%)	0.08	631 (58.6%)	1198 (41.9%)	< 0.001
Female		2067 (41.3%)	2173 (39.7%)		445 (41.4%)	1661 (58.1%)	
Routine haematological parameter							
Neutrophil percentage (NeuP, %)	45.0–77.0	63.6 [57.0, 70.6]	57.3 [52.1, 62.5]	< 0.001	61.6 [55.3, 68.2]	58.7 [52.2, 66.2]	< 0.001
Neutrophil count (NeuC, 10^9/L)	2.0–7.7	4.1 [3.2, 5.3]	3.1 [2.5, 3.8]	< 0.001	3.6 [2.9, 4.7]	3.4 [2.6, 4.3]	0.001
Monocytes percentage (MonP, %)	3.0–8.0	5.6 [4.5, 6.0]	5.0 [4.2, 6.0]	< 0.001	7.2 [6.1, 8.4]	7.0 [5.9, 8.3]	0.19
Mean corpuscular haemoglobin concentration (MCHC, g/L)	310.0–370.0	333.0 [324.0, 343.0]	330.0 [321.0, 341.0]	< 0.001	337.3 [331.3, 343.3]	335.9 [325.4, 346.9]	0.03
Lymphocyte percentage (LymP, %)	20.0–40.0	28.1 [21.5. 33.8]	34.5 [29.7, 39.5]	< 0.001	28.4 [22.2, 33.9]	31.1 [24.4, 38.3]	< 0.001
Red blood cell distribution width-CV (RDW-CV, %)	11.0–17.0	12.6 [12.0, 13.3]	13.0 [12.4, 13.6]	< 0.001	12.0 [11.3, 12.6]	12.3 [11.9, 13.0]	< 0.001
Mean corpuscular volume (MCV, fl)	86.0–100.0	92.2 [89.0, 96.0]	95.0 [91.0, 98.0]	< 0.001	91.0 [88.0, 94.0]	90.0 [86.9, 93.0]	0.36
Haemoglobin (Hgb, g/L)	110.0–160.0	137.0 [127.0, 147.0]	147.0 [137.0, 157.0]	< 0.001	130.0 [120.0, 141.0]	137.0 [126.0, 148.0]	< 0.001
Biochemical analyte							
Total cholesterol (TC, mmol/L)	3.0–6.5	4.7 [4.0, 5.4]	5.3 [4.7, 6.0]	< 0.001	4.4 [3.7, 5.3]	5.1 [4.4, 5.8]	< 0.001
High-density lipoprotein cholesterol (HDL-C, mmol/L)	0.9–2.2	1.2 [1.0, 1.4]	1.4 [1.2, 1.6]	< 0.001	1.1 [1.0, 1.3]	1.4 [1.1, 1.6]	< 0.001
Uric acid (UA, μmol/L)	90.0–420.0	299.0 [248.0, 357.0]	331.0 [278.0, 388.0]	< 0.001	280.0 [232.0, 332.5]	268.5 [217.0, 320.0]	0.002
Total protein (TP, g/L)	55.0–85.0	66.2 [62.2, 70.6]	70.6 [68.2, 73.2]	< 0.001	66.4 [62.2, 70.4]	72.6 [69.7. 75.8]	< 0.001
Calculated globulin (CG, g/L)	20.0–33.0	25.4 [22.7, 28.2]	27.3 [25.1, 29.5]	< 0.001	28.3 [25.6, 31.3]	30.1 [27.5, 32.7]	< 0.001
Alkaline phosphatase (AKP, U/L)	40.0–150.0	66.0 [55.0, 80.0]	63.0 [52.0, 74.0]	< 0.001	83.7 [69.0, 101.0]	84.8 [71.8, 102.8]	0.16

Continuous variables are presented as mean [IQR], categorical variable sex is presented as count (%). Mann–Whitney *U*-test and chi-squared test were performed for continuous and categorical variables respectively

### Selected features to develop machine learning-based prediction models

In total, 15 features were selected on training set: one demographic feature (age), eight routine haematological parameters (neutrophil percentage [NeuP], neutrophil count [NeuC], macrophage percentage [MonP], mean corpuscular haemoglobin concentration [MCHC], lymphocyte percentage [LymP], red blood cell distribution width-CV [RDW-CV], mean corpuscular volume [MCV], and haemoglobin [Hgb]), and six biochemical analytes (total cholesterol [TC], high density lipoprotein cholesterol [HDL-C], uric acid [UA], total protein [TP], calculated globulin [CG], and alkaline phosphatase [AKP]). Fig. S2 in Supplementary Information demonstrated 41 initial features and the results of the four feature selection approaches. The characteristics of the selected features and corresponding reference intervals are detailed in Table 1.

### Developing machine learning-based prediction models

Based on the selected features, supervised classification algorithms were applied to develop and compare six ML-based models. The models were fitted on the SAH-SFMU training dataset, and the optimised tunning parameters are provided in Table S1. Overall, the ML-based model is understandable to medical audiences making it feasible to convert into a usable prediction tool in clinical practice.

### Validating machine learning-based prediction models

**Fig. 2:**
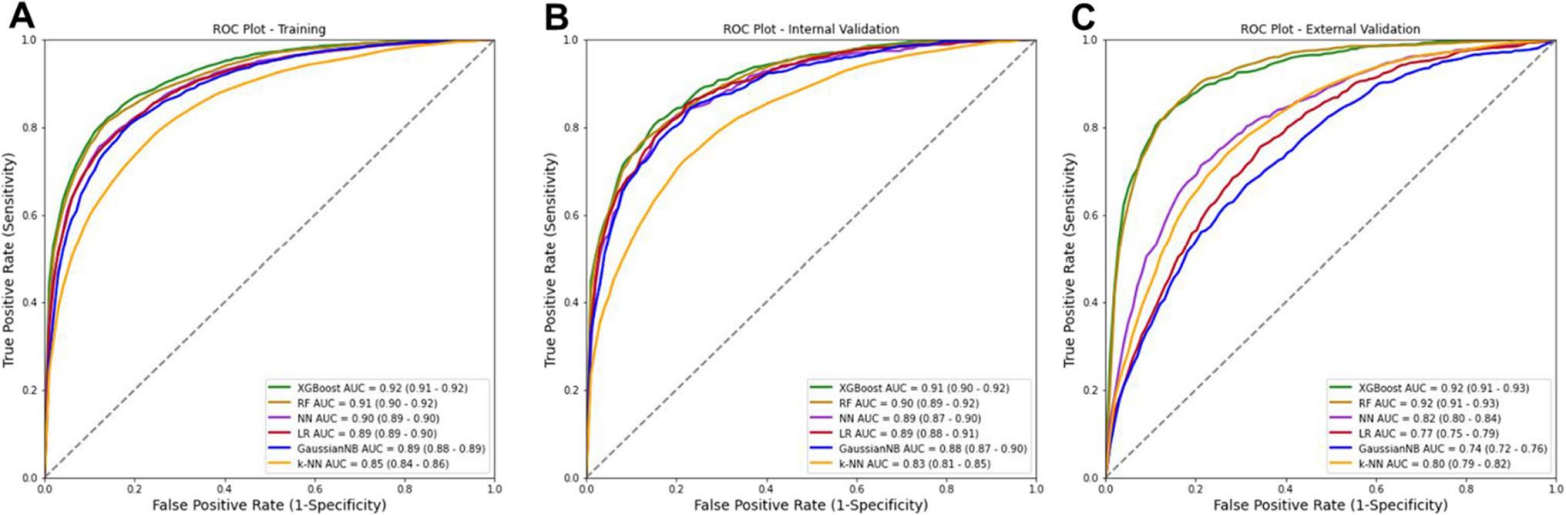
Comparison of the ROC curve of six machine learning-based models. **A-C** Performances for training, internal validation, and external validation sets. AUC value is obtained via the corresponding ML-based model, 95% AUC confidence intervals are presented in the parentheses. Abbreviations: ROC, receiver operating characteristic; AUC, area under the receiver operating characteristic curve; XGBoost, extreme gradient boosting; RF, random forest; NN, neural network; LR, logistic regression; GuaissianNB, Gaussian naive Bayes; k-NN, k-nearest neighbours

Established ML-based models were both internally validated using 20% of the SAH-SFMU data and externally validated using the entire DPH dataset. The results are incorporated into Fig. S3 and show the AUCs of the ML-based models in the fivefold cross-validation. The average AUC for each ML-based model was used as the primary performance metric in the training, internal validation, and external validation datasets (Fig. 2).

For triaging patients with IS, AUCs of the XGBoost-based model were 0.92 (95% CI 0.91–0.92) in the training dataset and 0.91 (0.90–0.92) in the internal validation dataset. When applied to the external validation dataset, the model yielded an AUC of 0.92 (0.91–0.93).

The XGBoost-based model achieved the highest average sensitivities (recalls): 0.81 in the training and internal validation datasets, and 0.71 in the external validation dataset. Whereas the GaussianNB-based model achieved the best specificity of 0.87 in the training and internal validation datasets, RF- and XGBoost-based models yielded the highest specificity in the external validation dataset.

Other performance metrics including classification accuracy, precision score, F1 score, and log loss, these results were in a relatively narrow range e.g. classification accuracies ranging from 0.77–0.84, 0.74–0.83, to 0.70–0.86 for the training, internal validation, and external validation datasets respectively. XGBoost-based model is the preferable model for screening IS patients by offering the highest sensitivity. Moreover, the XGBoost-based model had the lowest log loss value (0.36 for the training set, 0.38 for the internal validation set, and 0.33 for the external validation dataset), indicating that the XGBoost-based model has the lowest amount of uncertainty in the prediction. The performance of the ML-based models for the training, internal validation, and external validation datasets is described in Table 2.

**Table 2:** Summary of machine learning-based model’s performance in the training, internal validation, and external validation sets

	ML-based models	Sensitivity (recall)	Specificity	Classification accuracy	Precision score	F1 score	Log loss
Training	XGBoost	0.81	0.87	0.84	0.85	0.83	0.36
	RF	0.8	0.87	0.84	0.84	0.82	0.38
	NN	0.75	0.79	0.81	0.84	0.8	0.43
	LR	0.78	0.84	0.82	0.82	0.8	0.41
	GaussianNB	0.73	0.87	0.81	0.84	0.78	0.55
	k-NN	0.68	0.85	0.77	0.8	0.73	0.49
Internal validation	XGBoost	0.81	0.85	0.83	0.84	0.82	0.38
	RF	0.79	0.85	0.82	0.83	0.81	0.4
	NN	0.79	0.84	0.79	0.8	0.79	0.46
	LR	0.79	0.83	0.81	0.82	0.8	0.41
	GaussianNB	0.72	0.87	0.8	0.84	0.78	0.56
	k-NN	0.66	0.82	0.74	0.79	0.72	0.52
External validation	XGBoost	0.71	0.92	0.86	0.78	0.74	0.33
	RF	0.7	0.92	0.86	0.79	0.73	0.42
	NN	0.54	0.88	0.78	0.68	0.57	0.47
	LR	0.32	0.91	0.75	0.63	0.41	0.5
	GaussianNB	0.43	0.8	0.7	0.58	0.43	1.03
	k-NN	0.43	0.89	0.76	0.6	0.5	0.61

*XGBoost* extreme gradient boosting, *RF* random forest, *NN* neural network, *LR* logistic regression; *GuaissianNB* Gaussian naive Bayes, *k-NN* k-nearest neighbours

### Global and local model explanation

**Fig. 3:**
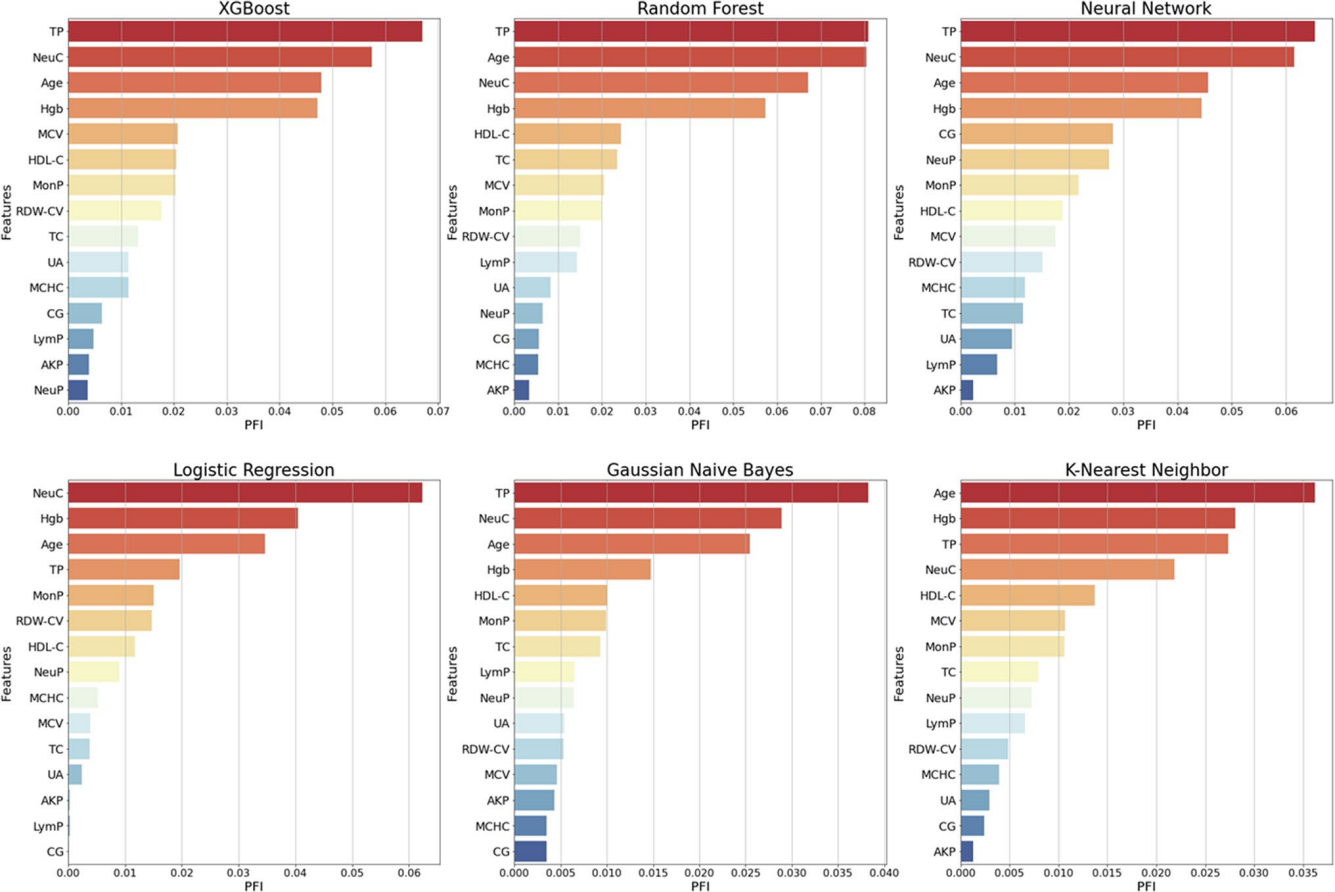
PFI of each machine learning-based model in the derivation dataset. Each histogram describes the PFI (also known as mean decrease accuracy) for a given ML-based model. The PFI is quantified by assigning the relative importance score for every independent input feature, indicating the relative importance of each feature when making a prediction. The top rankings are the most important features, while those bottom rankings matter least. Abbreviations: PFI, permutation feature importance; NeuP, neutrophil percentage; NeuC, neutrophil count; MonP, macrophage percentage; MCHC, mean corpuscular haemoglobin concentration; LymP, lymphocyte percentage; RDW-CV, red blood cell distribution width-CV; MCV, mean corpuscular volume; Hgb, haemoglobin; TC, total cholesterol; HDL-C, high-density lipoprotein cholesterol; UA, uric acid; TP, total protein; CG, calculated globulin; AKP, alkaline phosphatase

PFI technique was applied to explain how complex black-box ML-based models make predictions globally. By ranking the PFI score for each model, the 15 features were separated into two subgroups: strong informative vs. weak informative. The features allocated in the strong informative subgroup in this current study had vital influences on the predictability of the IS prediction model e.g. age, NeuC, TP, HDL-C, and Hgb are the most important features for IS prediction models. The other group included weaker features with less importance, such as AKP, CG, and LymP. Detailed PFIs and corresponding rankings for each model are demonstrated in Fig. 3*.*

**Fig. 4:**
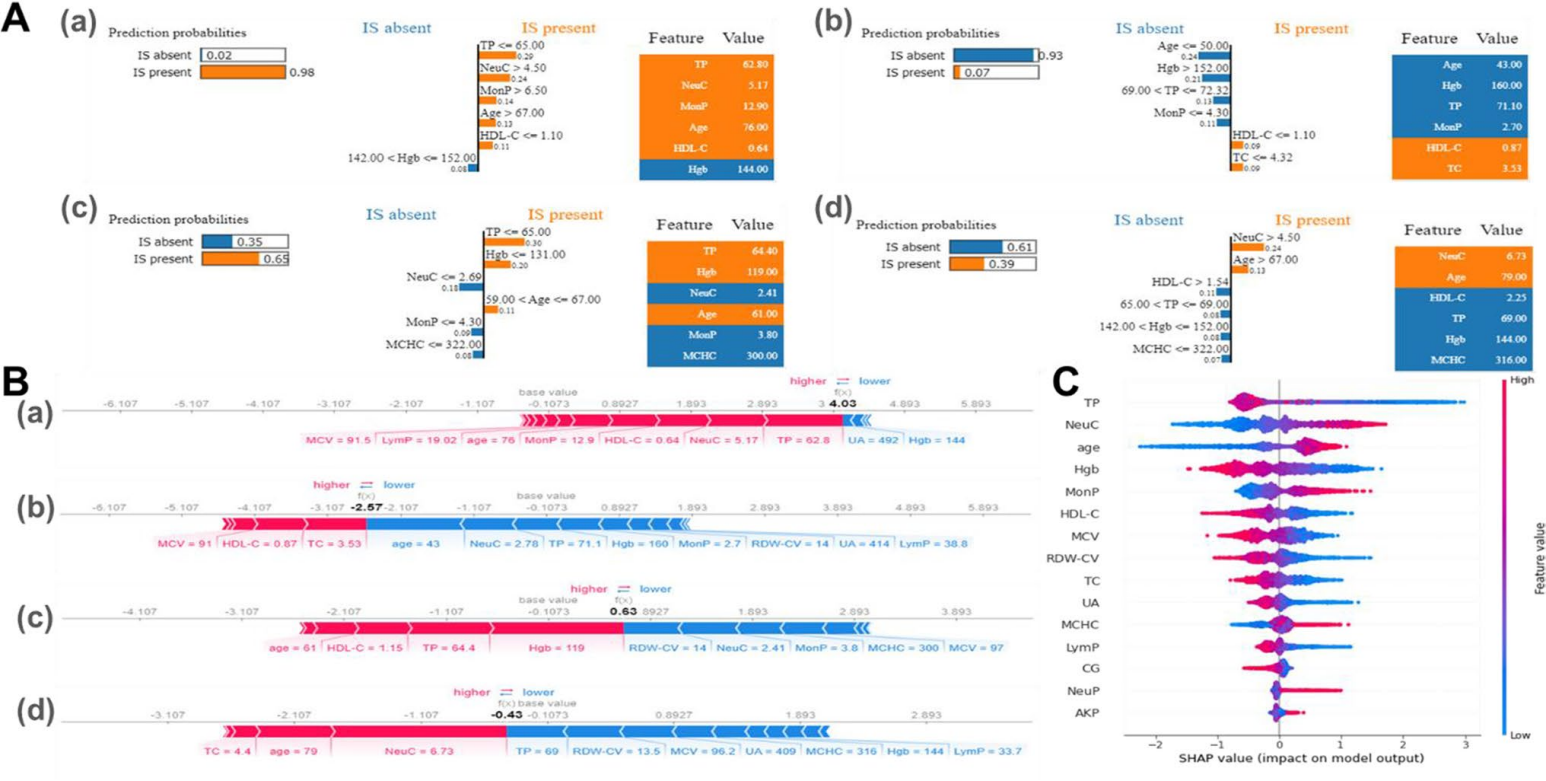
Interpretation of real-time sample prediction by LIME and SHAP. Explanations are based on the XGBoost model trained on the derivation dataset. (a–d) True positive, true negative, false positive, and false negative observations, respectively. **A** Four individual prediction scenarios through the LIME algorithm, orange features push the IS risk higher whereas blue features push the IS risk lower. **B** The four individual prediction scenarios through the XGBoost Tree SHAP algorithm. “Base value” marks the mean of the model output (log odds ratio) over the IS dataset; f(x) is the output value for a given observation; red arrows push the prediction towards high IS risk whereas blue arrows push towards low IS risk; the size of arrow marks the magnitude for the corresponding feature’s effect. **C** SHAP summary plot. Each dot represents a person in this study, the position of the dot on the *x-axis* indicates the feature impact on the model’s prediction for a specific person. The features listed on the *y-axis* are ordered based on their importance. Abbreviations: IS, ischemic stroke; LIME, local interpretable model-agnostic explanations; SHAP, SHapley Additive exPlanations; NeuP, neutrophil percentage; NeuC, neutrophil count; MonP, macrophage percentage; MCHC, mean corpuscular haemoglobin concentration; LymP, lymphocyte percentage; RDW-CV, red blood cell distribution width-CV; MCV, mean corpuscular volume; Hgb, haemoglobin; TC, total cholesterol; HDL-C, high-density lipoprotein cholesterol; UA, uric acid; TP, total protein; CG, calculated globulin; AKP, alkaline phosphatase

The case-level explanation involves the random drawing of the sample to make an individual prediction via the LIME algorithm and the XGBoost Tree SHAP algorithm [40]. The four individual real-time prediction scenarios (true positive, true negative, false positive, and false negative) are presented in Fig. 4 LIME is able to explain the model locally, explanations presented in Fig. 4A illustrating the complex XGBoost model could be described in an interpretable manner. For example, in Fig. 4A(a), this sample was rightly classified as an IS patient by the XGBoost model, the explanation assigns a weight of high IS risk to older age (67 years), low levels of HDL-C (1.1), TP (65.0) and high level of NeuC (4.5). These feature values are responsible for the XGBoost model’s IS-present prediction. For the SHAP method, the same individuals were selected as LIME, and local explanations offered by the SHAP force plot (Fig. 4B) were consistent with the explanations generated from LIME.

A SHAP summary plot was also provided to briefly display the direction and magnitude of a feature’s effect (Fig. 4C). It presents the direction of effects e.g. older age (red) had a higher IS risk than younger age (blue), as well as the distribution of effect sizes e.g. the long right tails for routine clinical test values. The long tails represent features with relatively low importance for the entire model that could be greatly important for single individuals. Moreover, the most important features determined by SHAP values (Fig. 4C) and by PFI (Fig. 3—XGBoost model) are the same.

### Implementation of the web-based tool

**Fig. 5:**
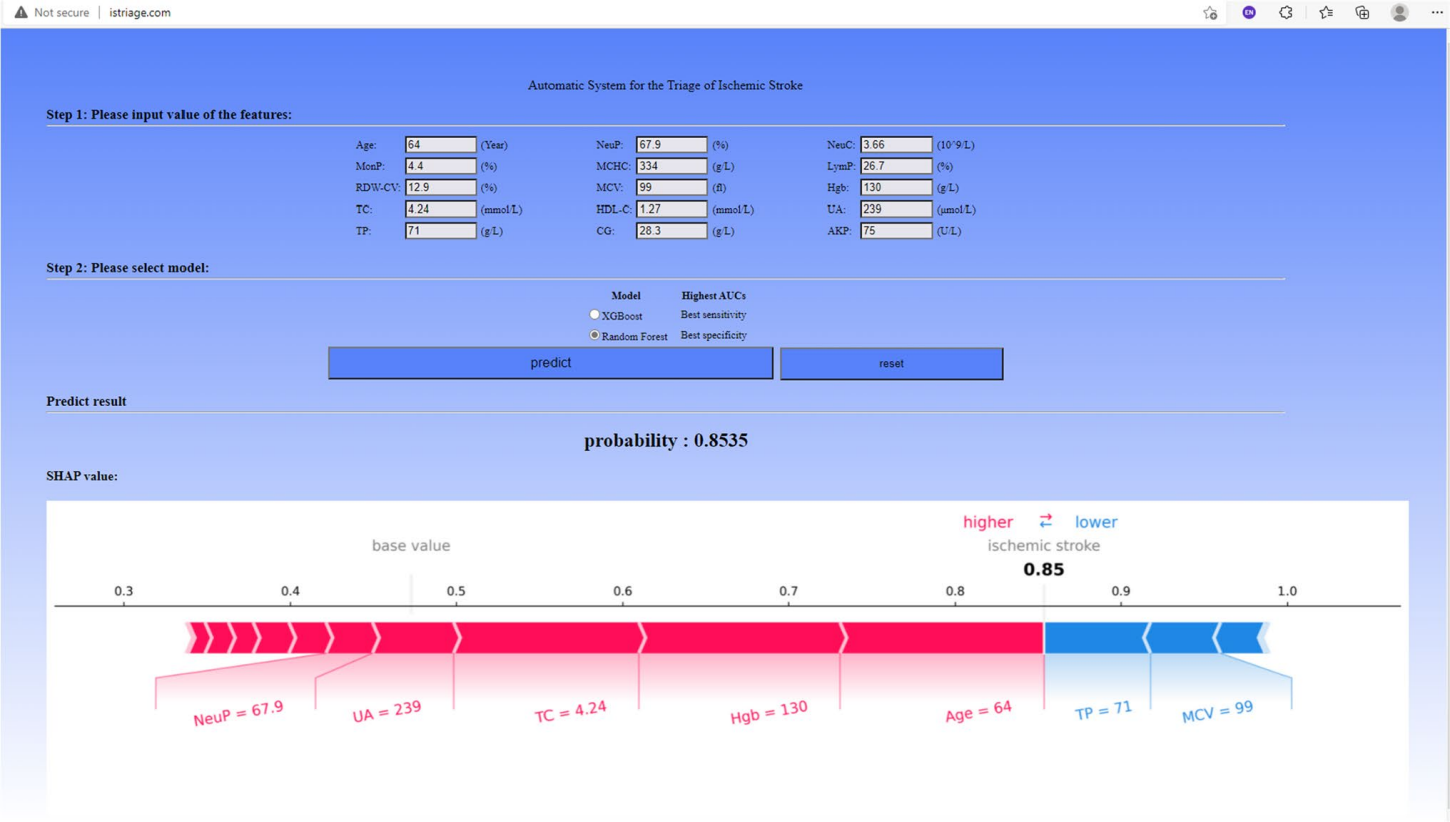
Website-Automatic System for the Triage of Ischemic Stroke. By inputting the example values of 15 clinical laboratory features and selecting the intended machine learning-based model, we can obtain a patient’s risk with ischemic stroke

We established a web-based tool (Automatic System for the Triage of Ischemic Stroke, ASTIS, [http://istriage.com/]) for clinical practice that can be widely applied in the evaluation of the risk for IS in primary care settings (Fig. 5). By entering the 15 clinical laboratory-related features and selecting the intended ML-based model, healthcare professionals could use ASTIS by visiting the website (http://istriage.com/) and obtain a rapid prediction for the IS. The higher the predicted probability, the higher the risk of the individual with suspected IS.

## Discussion

In this study, we considered the PPPM/3PM strategy for the diagnosis of IS and applied the cost-effective clinical laboratory patient profile to develop and validate the ML-based prediction models. We found that the selected 15 features presented optimal discriminative abilities for predicting the risk for IS. This outcome of the ML-based models was also externally validated in an independent dataset. Given the best AUCs and the highest sensitivities for internal and external datasets, the XGBoost-based model was therefore the preferred candidate model to identify the patients with IS in this current study. Key features for IS prediction were defined by measuring and ranking PFIs, and informative features included age, total protein, neutrophil count, and high-density lipoprotein cholesterol. The stability of the XGBoost model was verified through LIME and SHAP.

### ML-based models established with routine haematological and biochemical features bring broad translational applicability to the prediction of ischemic stroke

The paradigm shifts from the delayed reactive medicine to the proactive PPPM/3PM has been reckoned as a crucial transformation of the overall healthcare approach to benefit the patient and society at large [41]. Unlike conventional medical frameworks, the ML-based models for IS prediction in the PPPM/3PM framework could support the subjective decision making for healthcare providers, especially in resource-limited settings. This is beneficial for the patients’ stratification and the development of the individualised treatment.

Previous studies have identified predictive risk factors for stroke, the predictors/features are either costly or hardly obtained, especially in less-developed regions e.g. the biomarkers like cytokines and chemokines, or genetic predictors like circulating circular RNAs [42–44]. But in low-/middle-income regions, for example, in rural areas of China, patients normally face difficulty in accessing health facilities (such as CT and MRI). Since 2018, the *Standards of Service Capability of Community Health Service Centres and Township Health Centres* has been issued by China, and primary hospitals and healthcare centres are required to be equipped with basic medical services and health technologies, such as routine haematological and blood biochemistry tests [45]. Therefore, on top of the demographic information, identifying features that are easily available even in community or town hospitals plays a crucial role in developing rapid and accurate diagnostic tools to pre-screen the patients at risk for IS [46]. This can greatly support traditional physical examination for triaging patients with IS in resource-limited settings. Work on the Chinese Longitudinal Healthy Longevity Study (CLHLS) database demonstrated the feasibility of ML-based models using demographic and haematological features to predict stroke [8]. But such a viable approach for the early diagnostic screening of stroke has not yet been applied in clinical settings. This is mainly because of the complexity of the ML-based models, such as the XGBoost model and RF model, which are hard to be explained for patients and even for healthcare professionals.

### XGBoost-based model and explainable AI techniques enable precise and individualised predictions

Recent work showed AUCs of 0.65–0.78, sensitivities of 63–78%, and specificities of 60–79% in their internal validations [8, 47]. In contrast, the results in our current study showed higher applicability e.g. the XGBoost-based model developed in this study validates externally at a level of 0.92 (AUC) with routine haematological parameters and biochemical analytes considered, with sensitivities of 70% and specificities of 92%. Moreover, global and local explainable AI methods (PFI, LIME, and SHAP) can interpret how a complex black-box ML model makes a prediction. The developed XGBoost-based model in this study can be applied as a rapid triaging tool for pre-screening IS patients in less-developed regions with limited medical resources. This can reduce the delay in the referral of IS patients. The predictive analytic tool ASTIS (http://istriage.com/) was further implemented based on the established ML models and the SHAP technique, promising a quick and simple prediction for the risk of ischemic stroke.

### Recognition of age, neutrophil count, total protein, high-density lipoprotein cholesterol, and haemoglobin may help to enhance PPPM/3PM strategies to prevent IS

Identification of laboratory values plays an important role in developing the accurate prediction tool for IS, since the potential application is not only limited to diagnosis and clinical differentiation but also can be applied to the prognosis and patient monitoring [46, 47]. IS is a multi-factorial disease with both genetic and environmental aetiologies [2]. Over recent years, multi-omics projects involving genomics, transcriptomics, proteomics, glycomics, and metabolomics have offered an opportunity to understand the flow of information (e.g. the environmental risk) that underlies disease [48, 49]. To understand the complicated mechanisms underlying the IS in the future investigations, the different domains of the genetics, epigenetics, and environment should be considered; and their integrated effects on cerebrovascular health could contribute to the development of new prevention strategies and deeper insights into aetiological processes that lead to IS risk and susceptibility [3, 49].

Our current finding is consistent with prior research [50–57]. First, both human and animal studies revealed that ageing-related changes are associated with IS in terms of susceptibility, response to treatment, and prognosis [50]; relevant mechanisms may involve the accumulation of mitochondrial DNA, resulting in mitochondrial dysfunction which is associated with ageing-related neurological disorders, such as the pathological oxidative stress by ischemia–reperfusion damage [51, 52]. Second, a clinical trial and a review have revealed the potential mechanisms regarding the neutrophil count on IS risk, suggesting the interactions with the endothelium and platelets and overactivity of neutrophil extracellular traps may play a key role [53, 54]. Third, according to a bio-spectroscopic imaging investigation in IS, the level of total protein was found significantly reduced within the neuron soma and neuropil within the peri-infarct zone, indicating total protein is an effective predictor of IS risk [55]. Fourth, a prospective cohort study suggested a low HDL-C level in combination with a high TG level was associated with increased risks of ischemic stroke (in the current study, both HDL-C and TG have also been selected as features for the ML-based prediction model), particularly in those with other metabolic risk factors, such as high LDL-C level or with diabetes [56]; in a separate study, a significant lower level of HDL-C was observed in IS patients than in healthy controls, and this study additionally indicated that the specific N-glycosylation profile within immunoglobin G (IgG) may involve in pro-inflammatory IgG functionality and further lead to the pathogenesis of IS [3]. Last, a hospital-based cohort showed that lower haemoglobin levels are associated with larger stroke infarcts. Possible pathology demonstrated that haemoglobin is an essential oxygen-carrying molecule in vivo, and thus plays a crucial role in reducing the threshold for ischemia and resulting in higher IS risk [57]. In brief, the cost-effective routine haematological and biochemical features are reliable and quick for IS prediction when basic laboratory tests are available in the context of PPPM/3PM.

### Strengths and limitations

The advantage of this current investigation is that the datasets are non-synthetic which have higher distributions of IS cases and thus more likely to be objective and effective as a screening tool. Furthermore, ML algorithms avoid the overfitting phenomenon and perform well with feature selection, and adequate internal as well as external validation data to achieve a stable estimate. Fifteen easily accessible clinical laboratory features in this study could assist with the predictive diagnostics for suspected IS patients. In the context of PPM/3PM, this is an appropriate approach in IS management from the viewpoints of reasonability and cost-effectiveness.

However, there were also limitations. First, excluding patients younger than 40 would lead to models that would less likely detect stroke in the younger population. Second, the case–control ratio at relatively 1:1 may cause model performance that is different when models are used at the point of care. Further studies are needed to investigate any potential impact of false positives/negatives generated in real-world settings. Third, this prediction web tool is at a pilot stage and a qualitative assessment of the attitude of the healthcare providers towards this web tool is needed. To this end, we aim to apply our algorithm to the data which are generated from multi centres. Healthcare professionals could upload the patients’ demographic and real-time haematological or biochemistry features to the website (http://istriage.com/) developed by this current study and receive a real-time risk-factor analysis. The data could then be uploaded to a central database (e.g. in the cloud) which would be shared by their doctors.

## Conclusions and expert recommendations

In conclusion, we established and compared six ML-based models to pre-screen IS, showing that the XGBoost-based model has the highest overall predictive power, with AUC over 0.91 for the derivation dataset and external validation dataset. We also identified that age, NeuC, TP, HDL-C, and Hgb have important impacts/weights on the predictability of the models, while other predictors such as AKP, CG, and LymP are of less contribution to the prediction in the models. This study demonstrated that widely used clinical laboratory features supported by ML algorithms could serve as an effective triaging approach in targeting individuals with a high risk of IS (the implementation of ASTIS [http://istriage.com/]), particularly in the resource-limited primary healthcare settings.

This predictive analytic tool is quick and simple to administer but needs further calibration and validation in a longitudinal study. Further prospective data collection is needed using a cohort study design within real-world primary care settings, to confirm the validity of this current finding, and to further optimise the final risk prediction model. Overall, the application of ML-based models combined with explainable AI techniques facilitates the development of individualised predictive diagnostics, effective targeted prevention, and optimal treatments tailored to the personalised patient profile. This is supportive for the paradigm change from reactive medicine to PPPM/3PM.

## Electronic supplementary material

Below is the link to the electronic supplementary material.

Supplementary file1 (DOCX 2.51 MB)

## Data Availability

The data are available from the corresponding authors on a reasonable request.

## Code availability

The code used in this study is available from the corresponding author on reasonable request.
