# Supplementary material for: Rapid triage for ischemic stroke: a machine learning-driven approach in the context of predictive, preventive and personalised medicine
Source: EPMA J. 2022 May 27;13(2):285–98. doi: 10.1007/s13167-022-00283-4 (PMC9203613; doi:10.1007/s13167-022-00283-4)
Supplement: Supplementary file 1 — Supplementary file1 (DOCX 2.51 MB) [file 13167_2022_283_MOESM1_ESM.docx]

**Supplementary Information**

**Rapid triage for ischemic stroke: a machine learning-driven approach in the context of predictive, preventive and personalized medicine**

Yulu Zheng^1†^, Zheng Guo^1†^, Yanbo Zhang^2†^, Jianjing Shang^3^, Leilei Yu^4^, Ping Fu^5^, Yizhi Liu^6^, Xingang Li^1^, Hao Wang^7,8^, Ling Ren^9^, Wei Zhang^10^, Haifeng Hou^6,2,1*^, Xuerui Tan^11*^, Wei Wang^1^^,6,8,11,12*^, on behalf of Global Health Epidemiology Reference Group (GHERG)

**Machine learning algorithms**

In this work, the performances of six different machine learning methods were compared: LR (logistic regression), k-NN (k-nearest neighbour), RF (random forest), XGBoost (extreme gradient boosting), NN (neural network) and GaussianNB (Gaussian distribution Naive Bayes classifier).

(1) Logistic regression (LR) is a form of binomial regression when the outcome variable of interest is dichotomous [1]. (Eq.1)

$logit \left( P \right)=\ln\left( \frac{P}{1-P} \right)= \beta0+ \beta1X1+ \beta2X2+ \ldots+ \beta mXm$ (1)

Where *P* is the probability, β_0_ is the constant, β_j_ represents the partial regression coefficient for the corresponding independent variable X_j_.

(2) K-nearest neighbour (K-NN) is a non-parametric and instance-based machine learning algorithm can be used in both classification and regression problems. In this work, k-NN is applied to define the class [2]. (Eq.2)

$y ̂( x)=\frac{1}{k} \sum xi\in Nk (x)yi$ (2)

Where N_k_ (X) represents k nearest points to variable x for training data.

(3) Random forest (RF) customises two techniques: 1) classification technique and 2) regression tree (CART) technique. These techniques progress uncorrelated combination or multiple decision trees cantered on bootstrap aggregation (bagging) technique [3]. RF selects a subset randomly to build an independent decision tree. It is a repetition process that splits the selected random subset from the root node to a child node. This splitting continues till each tree reaches a leaf node without cropping. Each tree makes the classification of the features and the objective variable independently and votes for the final tree class. The overall classification is based on the majority acquired trees voting [4].

(4) XGBoost is a scalable tree-based gradient boosting algorithm. It utilized second-order Taylor expansion of the loss function [5]. (Eq.3)

$Obj=\sum_{i=1}^{n} l \left( y_{i},\hat{y}_{i} \right)+\sum_{k=1}^{k} \Omega\left( f_{k} \right)$ (3)

Where Obj is the objective function of XGBoost, k represents the number of trees (n_estimators), $\sum_{i=1}^{n} l \left( y_{i},\hat{y}_{i} \right)$ represents the training loss, and $\sum_{k=1}^{k} \Omega\left( f_{k} \right)$ is the model complexity.

$f_{t} \left( \chi_{i} \right)= w_{q}(x_{i})$ (3.1)

Specifically, we use $q(x_{i})$ to represent the leaf node where sample $x_{i}$ is located, additionally $w_{q}(x_{i})$ is used to represent the score obtained by this sample falling on the $q(x_{i})$ leaf node in binary search tree (t).

$\Omega\left( f_{t} \right)=\gamma T+\frac{1}{2}\lambda\sum_{j=1}^{T} w_{j}^{2}$ (3.2)

Then, T represents the number of leaf nodes, w is the vector of leaf nodes, γ denotes the difficulty degree of splitting nodes and λ is the L2 regularization coefficient.

(5) Neural network (NN) structure is split into layers of multiple nodes; data travels from the input layer and reaches the output layer after passing through hidden layers, each of the layers transforms the data into relevant information and provides the desired output [6]. (Eq.4)

$Y=f\left( b+\sum_{i=1}^{n} w_{i}x_{i} \right)$ (4)

Where Y is output of neuron, the function f is non-linear and is called the Activation Function, b is the bias, w represents the weights associated with the inputs.

(6) Gaussian distribution Naive Bayes classifier (GaussianNB) supports continuous valued features and models each as conforming to a Gaussian (normal) distribution [7]. (Eq.5)

$P_{\left( \left. x_{i} \right|y \right)}=\frac{1}{\sqrt{2\pi\sigma_{y}^{2}}}\exp\left( -\frac{\left( x_{i}-\mu_{y} \right)^{2}}{2\sigma_{y}^{2}} \right)$ (5)

Where $P_{\left( \left. x_{i} \right|y \right)}$ for obtaining the conditional probabilities of a test feature given a class and $x_{i}$ is a test data feature, $y$ is a class, and $\sigma_{y}^{2}$ is the associated sample variance.

**Ways and metrics for model evaluation**

(1)-(5) are common terms for a binary classification, and (6)-(14) are evaluation metrics to assess the performance of each model.

1. ***True Positive (TP)***, the number of cases which were predicted to be a **Positive** value and were actually observed to be a **Positive** value.
2. ***True Negative (TN)***, the number of cases which were predicted to be a **Negative** value and actually were observed to be a **Negative** value.
3. ***False Positive (FP)***, the number of cases which were predicted to be a **Positive** value but were actually observed to be a **Negative** value (Type I error).
4. ***False Negative (FN)***, the number of cases which were predicted to be a **Negative** value but were actually observed to be a **Positive** value (Type II error).
5. ***Confusion matrix*** was used to describe the performance of a classification model (or "classifier"). (Box S1)

**Box S1. Confusion matrix**

| **Confusion Matrix** | **Predicted**  **Negative** | **Predicted**  **Positive** |
| --- | --- | --- |
| **Actual Negative** | TN | FP |
| **Actual Positive** | FN | TP |

1. ***Classification accuracy***, the percentage of true outcomes among the total number of cases tested. (Eq.6)

$Accuracy=\frac{TP+TN}{TP+TN+FP+FN}$ (6)

1. ***True Positive Rate (TPR),*** *also known as* ***Recall*** *or* ***Sensitivity***, the proportion of actual Positives is correctly classified. (Eq.7)

$TPR=Recall=Sensitivity=\frac{\mathrm{TP}}{TP+FN}$ (7)

1. ***True Negative Rate (TNR),*** *or* ***Specificity***, the proportion of actual Positives is correctly classified. (Eq.8)

$TNR=Specifity=\frac{\mathrm{TN}}{TN+FP}$ (8)

1. ***False Positive Rate (FPR),*** *or* ***type I error probability***. (Eq.9)

$FPR=1-Specificity=\frac{\mathrm{FP}}{TN+FP}$ (9)

1. ***Precision***, the proportion of predicted Positives is truly Positive. (Eq.10)

$Precision=\frac{\mathrm{TP}}{TP+FP}$ (10)

1. ***F1 score***, also known as ***balanced*** ***F-score*** or ***F-measure***. It is a number between 0 and 1 and can be interpreted as weighted average pf the precision and recall. (Eq.11)

$F_{1}=2*\frac{Precision*Recall}{Precision+Recal}$ (11)

1. ***Log loss,*** also known as ***logistic*** ***loss*** or ***cross-entropy*** ***loss***, is only defined for two or more labels. (Equ.12)

$L\log\left( y,p \right)=-\left( y\log\left( p \right)+\left( 1-y \right)\log\left( 1-p \right) \right)$ (12)

Where *y* is a true label in this binary classification, *L* is loss function, *p* is the probability estimate.

(13) ***Receiver Operating Characteristic (ROC) curve***, a curve determined by TPR and FPR for evaluating the model performance.

(14) ***Area Under Curve (AUC)*** is the area under the ROC curve.

**Supplementary figures and tables**

**
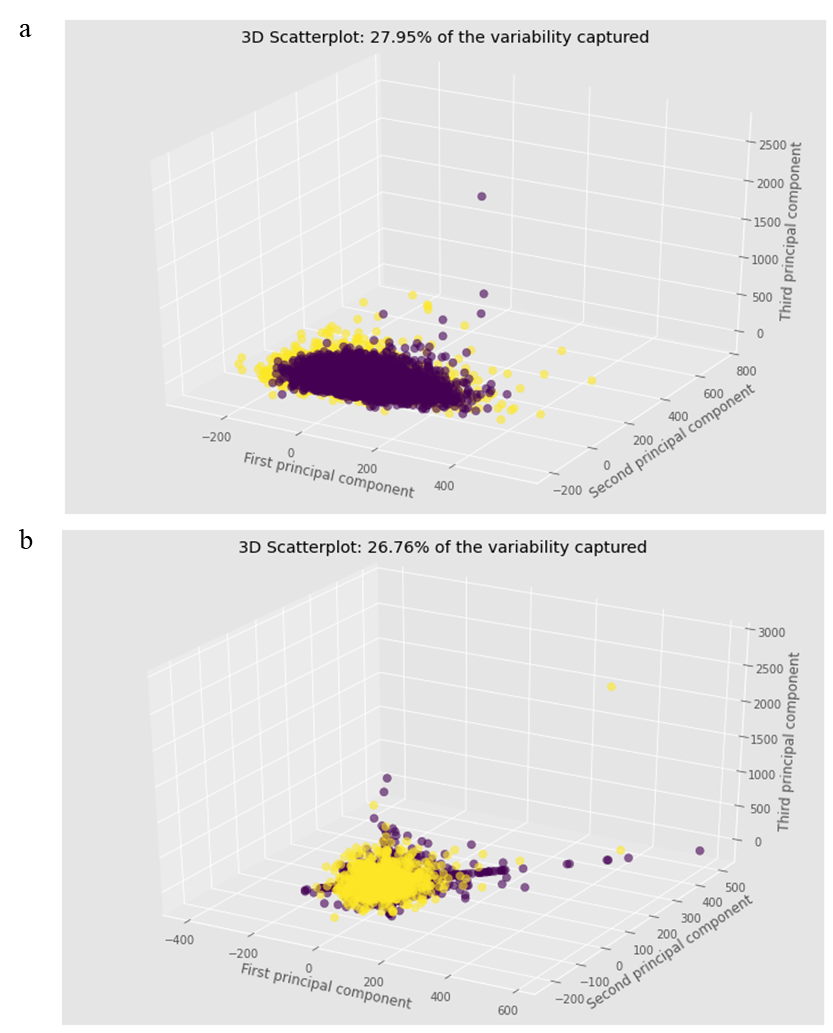
**

**Fig. S1.** **Three component principal component analysis (PCA) plots.** **a.** PCA plot for the derivation dataset. **b.** PCA plot for the external validation dataset. The yellow dot represents the samples without IS, and the purple triangle represents the samples with IS. The three principal components explained 27.95% and 26.76% of the observed variance respectively for the derivation dataset and the external validation dataset. *Abbreviations*: IS, ischemic stroke; PC, principal component.


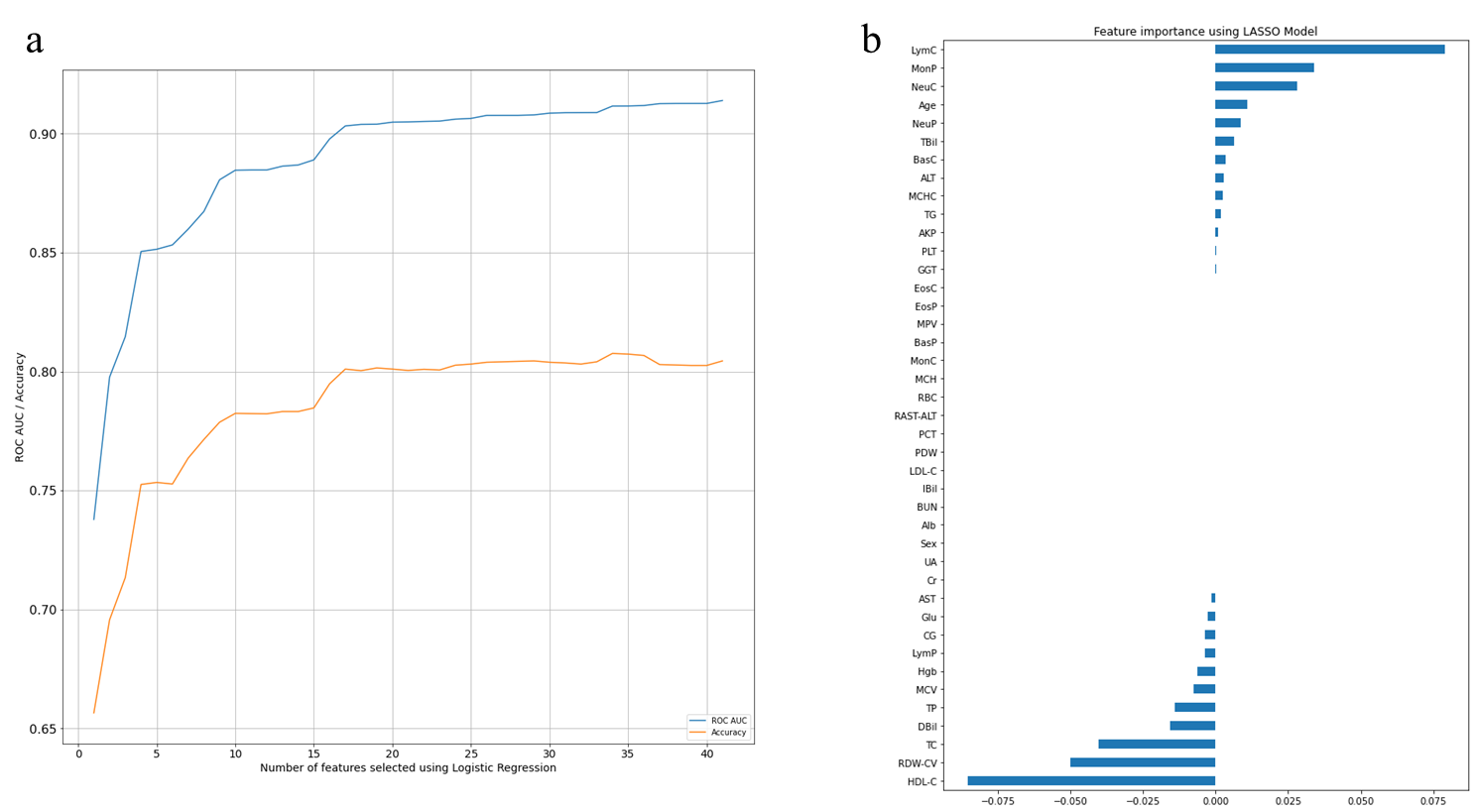


**Fig. S2. Results of four feature selection methods.** *Continued*

**
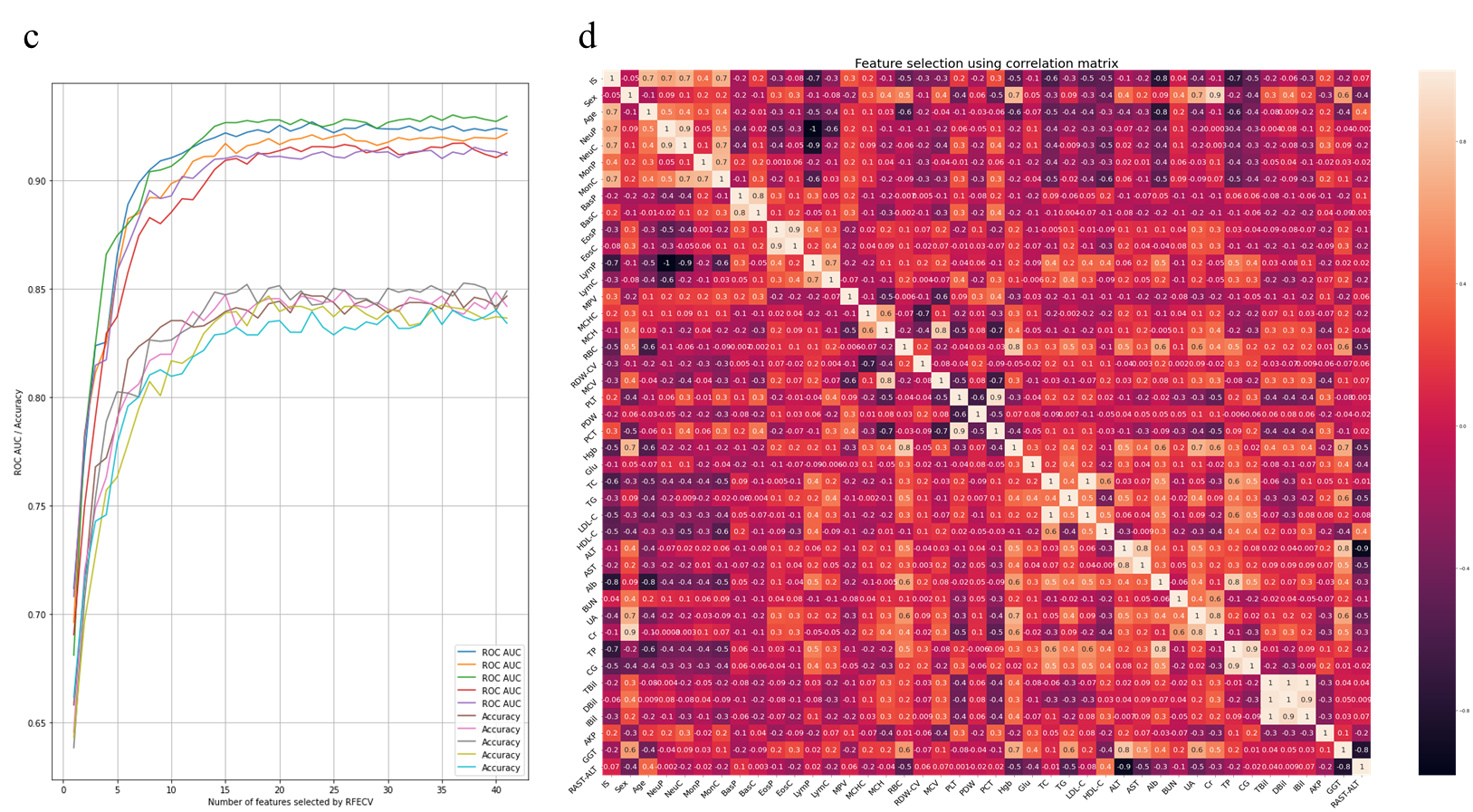
**

**Fig. S2. Results of four feature selection methods. a** Logistic regression. **b** Least absolute shrinkage and selection operator (LASSO) regression. **c** Recursive feature elimination with 5-fold cross-validation (RFECV). **d** Spearman correlation. For **a** and **c**, the model’s AUC and accuracy values (Y-axis) is compared to the number of features used in the model (X-axis) within the SAH-SFMU training dataset. In **c**, increasing the number of features strengthens the model to a plateau point which is reached around 15 features. 41 initial features included Age, Sex, Neutrophil percentage (NeuP, %), Neutrophil count (NeuC, 10^9/L), Monocytes percentage (MonP, %), Monocyte count (MonC, 10^9/L), Basophils percentage (BasP, %), Basophil count (BasC, 10^7/L), Eosinophils percentage (EosP, %), Eosinophil count (EosC, 10^9/L), Mean platelet volume (MPV, fl), Mean corpuscular haemoglobin concentration (MCHC, g/L), Mean corpuscular haemoglobin (MCH, pg), Lymphocyte percentage (LymP, %), Lymphocyte count (LymC, 10^9/L), Red blood cells (RBC, 10^12/L), Red blood cell distribution width-CV (RDW-CV, %), Mean corpuscular volume (MCV, fl), Platelet (PLT, 10^9/L), Platelet distribution width (PDW, %), Plateletcrit (PCT, %), Haemoglobin (Hgb, g/L), Glucose (Glu, mmol/L), Total cholesterol (TC, mmol/L), Triglyceride (TG, mmol/L), Low density lipoprotein cholesterol (LDL-C, mmol/L), High density lipoprotein cholesterol (HDL-C, mmol/L), Blood urea nitrogen (BUN, mmol/L), Uric acid (UA, μmol/L), Creatinine (Cr, μmol/L), Total protein (TP, g/L), Calculated globulin (CG, g/L), Albumin (Alb, g/L), Alkaline phosphatase (AKP, U/L), Glutamyl transpeptidase (GGT, U/L), Total bilirubin (TBil, μmol/L), Direct bilirubin (DBil, μmol/L), Indirect bilirubin (IBil, μmol/L), Aspartate transaminase (AST, U/L), Alanine aminotransferase (ALT, U/L), Ratio of AST and ALT (RAST-ALT).

**Table S1. Tuning parameters for six predictive models within the SAH-SFMU training dataset**

| ML-based Models | Tunning | Optimized tuning parameters |
| --- | --- | --- |
| XGBoost | Depth (maximum depth of number), weight (the smallest | max_depth = 5, |
|  | sample weight and weight in the child node) | min_child_weight = 1 |
| RF | Subtrees (# n_estimators) | n_estimators = 198 |
| NN | α (Regularization parameter), | α = 0.01, |
|  | size (#hidden layer units) | hidden_layer_size = 19 |
| LR | C (inverse of regularization strength) | C = 0.21 |
| GaussianNB | Priors (prior probabilities of the classes) | priors = None |
| k-NN | k (#Neighbors) | n_neighbors = 31 |

*Abbreviations*: SAH-SFMU, the Second Affiliated Hospital of Shandong First Medical University; ML, machine learning; XGBoost, extreme gradient boosting; RF, random forest; NN, neural network; LR, logistic regression; GuaissianNB, Gaussian naive Bayes; k-NN, k-nearest neighbours.

**
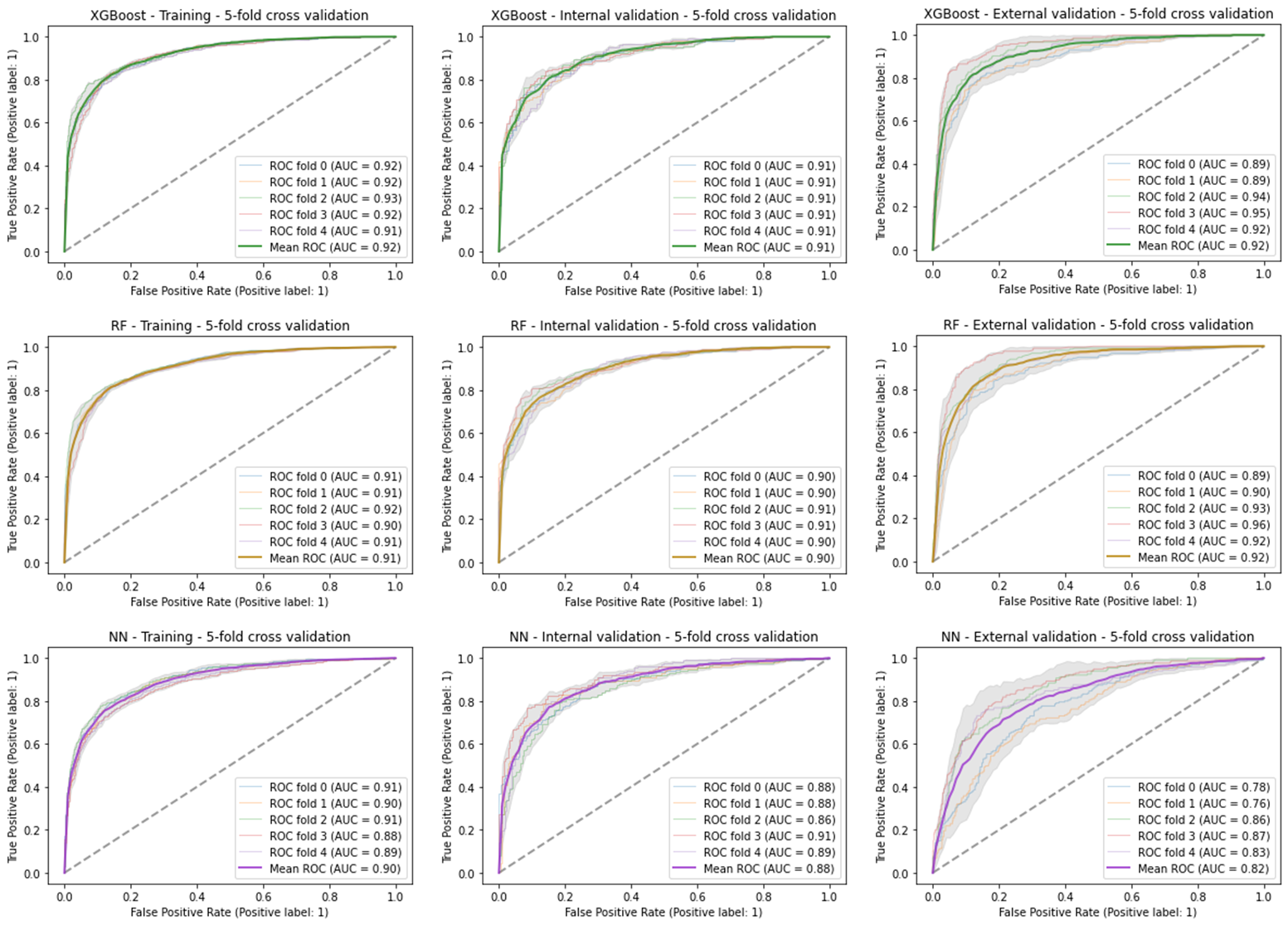
**

**Fig. S3. AUCs of machine learning-based models in the 5-fold stratified cross-validation.** *Continued*

**
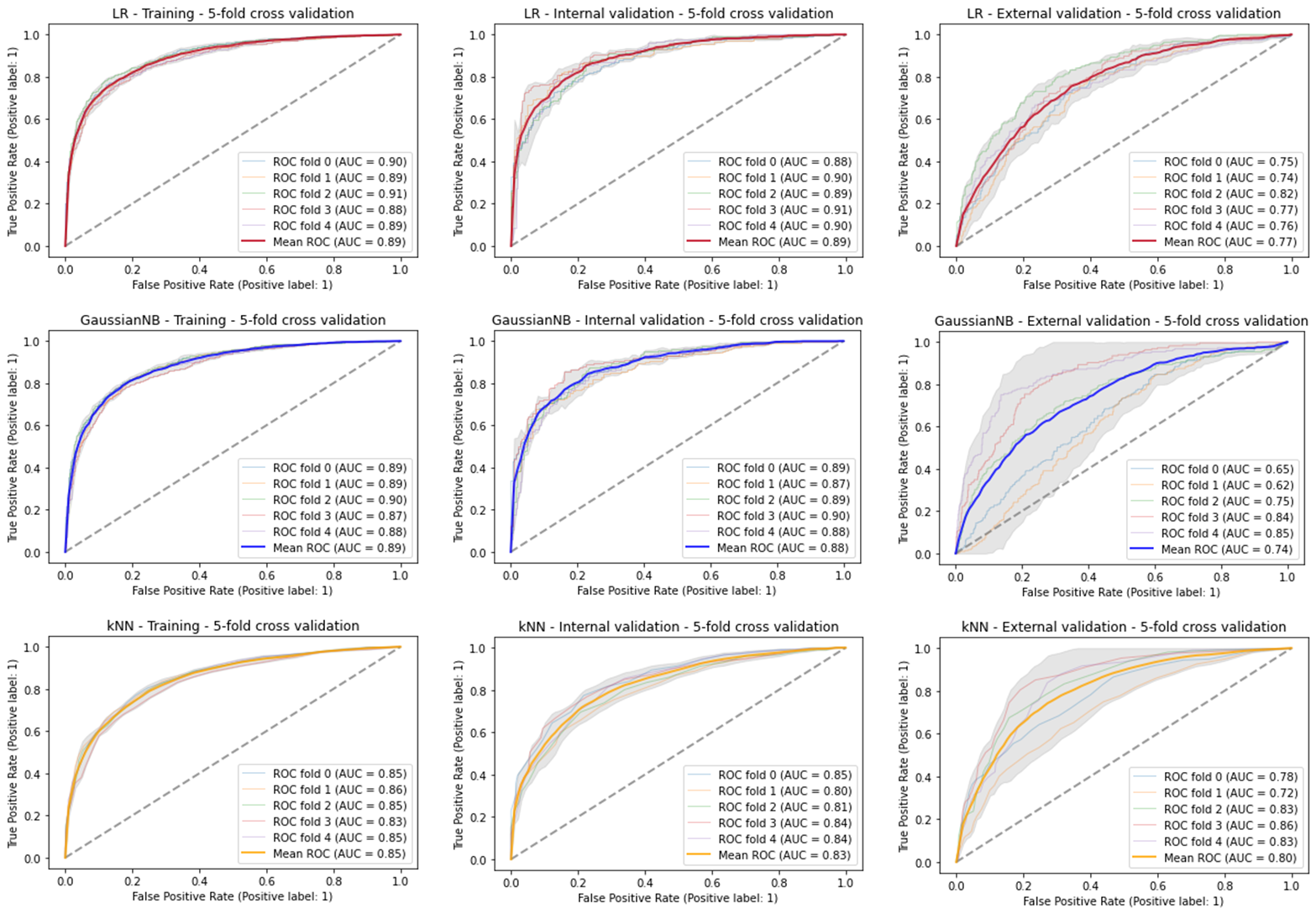
**

**Fig. S3. AUCs of machine learning-based models in the 5-fold stratified cross-validation**. ROC curves for six machine learning-based models respectively on first-sixth rows. Train, internal validation, and external validation performances are reported respectively on first, second and third columns. Six machine learning-based models are compared.

**References**

1. Chen T-H, Chen C-Y, Yang H-CP, Chen C-W. A mathematical tool for inference in logistic regression with small-sized data sets: a practical application on ISW-ridge relationships. Mathematical Problems in Engineering. 2008;2008:1-13.

2. Dubey H, Pudi V, editors. Class based weighted k-nearest neighbor over imbalance dataset. Pacific-Asia conference on knowledge discovery and data mining; 2013: Springer.

3. Liaw A, Wiener M. Classification and regression by randomForest. R news. 2002;2(3):18-22.

4. Rodriguez-Galiano V, Sanchez-Castillo M, Chica-Olmo M, Chica-Rivas M. Machine learning predictive models for mineral prospectivity: An evaluation of neural networks, random forest, regression trees and support vector machines. Ore Geology Reviews. 2015;71:804-18.

5. Ge Y, Wang Q, Wang L, Wu H, Peng C, Wang J, et al. Predicting post-stroke pneumonia using deep neural network approaches. International journal of medical informatics. 2019;132:103986.

6. Kaur H, Kumari V. Predictive modelling and analytics for diabetes using a machine learning approach. Applied computing informatics. 2020:1-6.

7. Ali L, Khan SU, Golilarz NA, Yakubu I, Qasim I, Noor A, et al. A feature-driven decision support system for heart failure prediction based on statistical model and Gaussian naive bayes. Computational Mathematical Methods in Medicine. 2019;2019:1-9.
